# A165 VEDOLIZUMAB IS AN OPTION TO TREAT MICROSCOPIC COLITIS IN PATIENTS DEPENDENT ON ORAL STEROIDS OR HAVE FAILED A BIOLOGIC: A MULTICENTRE CANADIAN CASE SERIES

**DOI:** 10.1093/jcag/gwab049.164

**Published:** 2022-02-21

**Authors:** A Kulyk, K Muthiah

**Affiliations:** Internal Medicine, University of Manitoba, Winnipeg, MB, Canada

## Abstract

**Background:**

Microscopic colitis (MC) is an inflammatory bowel condition demonstrating normal mucosa on endoscopy, with evidence of inflammation and lymphocytosis on pathologic analysis of biopsies. The pathophysiology for this disease is largely unknown, and evidence for its management in cases refractory to oral steroids or after failing a biologic is sparse. Vedolizumab, an integrin receptor antagonist, has been used in a handful of persons with MC with limited literature.

**Aims:**

Review literature on the use of Vedolizumab in treatment of MC. Present 7 cases of MC where Vedoluzimab was used to manage patients refractory to therapy (either steroid dependent, or failing biologic therapy).

**Methods:**

Patients with MC managed with Vedolizumab were identified by soliciting practicing Gastroenterologists within Winnipeg at 3 health centres. A chart review was conducted to obtain pertinent information including patient demographics, symptoms, and medications previously trialed.

**Results:**

Review of literature revealed 3 case reports, and 4 case series reporting on the use of Vedolizumab in MC. We identified 7 cases; 6 female, and a mean age at diagnosis of 50.7. Histologies included 3 lymphocytic, 2 collagenous, 1 developed from lymphocytic to collagenous over time, and 1 with evidence of both. 4 patients were on a biologic prior to initiating Vedolizumab with a mean duration of therapy of 24.25 months. Indications for initiating Vedolizumab included: 3 with steroid dependency, 3 Infliximab failures, and 1 with Adalimumab-associated hepatotoxicity. Mean disease duration prior to initiating Vedolizumab was 112.7 months. Number of stools pre-Vedolizumab were: 1–3 = 0, 4–6 = 2, 7–9 = 2, >/=10 = 3, compared to >6 months post-Vedolizumab: 1–3 = 5, 4–6 = 1, 7–9 = 1, >/=10 = 0. Symptoms pre-Vedolizumab compared to >6 months post-Vedolizumab included: 4 with urgency pre-Vedolizumab versus 1, 3 had incontinence versus 0, and 4 had nocturnal symptoms versus 0. Use of alternative medications included anti-diarrheals, oral 5-aminosalicylic acid, and immunomodulators. On average, patients tried 7.6 different treatments prior to starting Vedolizumab. 1 case is considered to have failed Vedolizumab due to increasing symptoms.

**Conclusions:**

This multicentre case series is the first in Canada to assess patient characteristics and symptoms before and after Vedolizumab induction in persons with MC who are either steroid dependent or have failed a biologic. Our results demonstrate an overall improvement in symptoms when assessed >6 months after induction therapy. This review was limited in not having a symptom inventory/severity index completed at each clinic visit, resulting in a number of difficult-to-compare and incomplete data points.

**Funding Agencies:**

None

